# Psychotherapie in der stationären Psychiatrie: von der Personalausstattung zur Patientenkontaktzeit

**DOI:** 10.1007/s00115-026-01939-6

**Published:** 2026-01-26

**Authors:** Johannes Kornhuber, Manuel Maler, Timo Oberstein

**Affiliations:** Psychiatrische und Psychotherapeutische Klinik, Friedrich-Alexander-Universität Erlangen-Nürnberg (FAU), Schwabachanlage 6, 91054 Erlangen, Deutschland

## Hintergrund

Die Studie der Bundespsychotherapeutenkammer (BPtK) zur psychotherapeutischen Versorgung in der stationären Psychiatrie [1] hat eine breite Diskussion ausgelöst, die auch die *Süddeutsche Zeitung* aufgegriffen hat [2]. Die Daten zeigen: Stationäre Patienten erhalten durchschnittlich nur 25 min Einzelpsychotherapie pro Woche – als Summe aus ärztlicher und psychologischer Therapie mit mindestens 25 min Dauer.

Das psychiatrische Fach sollte die Meinungsführerschaft in dieser Diskussion übernehmen und konstruktive Lösungen aufzeigen. 50 Jahre nach der Psychiatrie-Enquete haben wir die personellen Voraussetzungen verbessert – nun gilt es, diese optimal zu nutzen. Dabei muss klar benannt werden: Für eine vollständig leitliniengerechte Behandlung reicht die Optimierung vorhandener Ressourcen nicht aus – hierfür wäre eine substanzielle Erhöhung der Personalvorgaben erforderlich. Der vorliegende Beitrag zeigt, welche Verbesserungen mit den aktuellen Ressourcen erreichbar sind und wo die strukturellen Grenzen liegen. Nach der Einreichung dieses Beitrags veröffentlichte die Deutsche Gesellschaft für Psychiatrie und Psychotherapie, Psychosomatik und Nervenheilkunde e. V. (DGPPN) eine Stellungnahme, die das BPtK-Papier als methodisch unvollständig kritisiert, da Gruppentherapien und psychosoziale Therapien nicht erfasst wurden [3]. Diese Kritik greift jedoch zu kurz: Die Netzwerkmetaanalyse von Mavranezouli et al. zeigt gerade für schwere Depressionen, dass Einzeltherapien wirksam sind, Gruppentherapien jedoch nicht [4]. Zudem adressiert die DGPPN-Stellungnahme nicht die zentrale Frage, wie die vorhandenen Personalressourcen optimal für den direkten Patientenkontakt eingesetzt werden können. Genau hier setzt der vorliegende Beitrag an.

## Die Datenlage

Die BPtK-Studie dokumentiert die tatsächlich erbrachte Einzelpsychotherapie [1]. Ärzte erbringen durchschnittlich 11,4 min Einzeltherapien pro Woche mit mindestens 25 min Dauer, Psychologen etwa 13,5 min; in Summe knapp 25 min als langfristiger Mittelwert: Einige Kliniken bieten mehr, andere weniger. In knapp der Hälfte aller Behandlungswochen findet keine solche Einzeltherapie statt – trotz einer durchschnittlichen Personalausstattung von 120 % der Vorgaben der Personalausstattung Psychiatrie und Psychosomatik-Richtlinie (PPP-RL).

Kürzere Gespräche und Gruppentherapien wurden nicht erfasst – diese können im Einzelfall durchaus hilfreich sein. Dennoch bleibt bemerkenswert, dass ein Mindestmaß an Einzelgesprächen mit ausreichender Dauer offenbar nicht durchgängig gewährleistet ist.

Noch bemerkenswerter ist die Diskrepanz zur subjektiven Wahrnehmung: Der Abschlussbericht des IGES Instituts zur PPP-RL zeigt eine deutliche Überschätzung der tatsächlich erbrachten Therapie durch Klinikleitungen [5]. Diese fehlende Problemwahrnehmung ist entscheidend: Was nicht wahrgenommen wird, wird auch nicht verbessert.

## Die Evidenzbasis

Die Netzwerkmetaanalyse von Mavranezouli und Kollegen zeigt für schwere Depressionen, dass Einzeltherapien wirksam sind, Gruppentherapien dagegen nicht [4]. Schwer depressive Patienten mit sozialem Rückzug, kognitiven Einschränkungen und reduziertem Selbstvertrauen werden in Einzeltherapien meist besser erreicht als in Gruppensettings. Eine Metaregressionsanalyse von Ciharova und Kollegen belegt zudem, dass häufigere Psychotherapie – mehr als eine Sitzung pro Woche – bessere Ergebnisse erbringt als seltenere Kontakte [6].

Expertengruppen haben auf Basis der S3-Leitlinien konkrete Empfehlungen erarbeitet: Für unipolare Depressionen werden 100 min Einzelpsychotherapie pro Woche empfohlen [7]. Für die chronische Depression liegt eine differenzierte Analyse vor [8]. Diese Empfehlungen liegen um ein Mehrfaches über den derzeit erbrachten 25 min pro Woche. Wir haben uns hier auf die Depression beschränkt; die Expertenempfehlungen für Schizophrenien oder Angsterkrankungen liegen sogar noch höher.

## Die PPP-RL richtig verstehen

Die PPP-RL definiert die Mindestvoraussetzungen zur Sicherstellung der Grundversorgung – sie ist kein Personalbemessungsinstrument für leitliniengerechte Therapie. Samaan und Kollegen haben berechnet, dass für eine vollständig leitliniengerechte Behandlung ein Mehrbedarf von etwa 100 % im ärztlichen und psychologischen Bereich erforderlich wäre [7]. Eine Optimierung vorhandener Ressourcen kann die Versorgung erheblich verbessern, aber die vollständige Umsetzung der Leitlinienempfehlungen erfordert zusätzlich eine Anpassung der Personalvorgaben.

## Das Konzept: On-Zeit und Off-Zeit

Wie erklärt sich das Defizit an Einzelpsychotherapie? Die PPP-RL betrachtet die Anwesenheit von Mitarbeitern in der Klinik, definiert aber nicht, welcher Zeitanteil für direkten Patientenkontakt vorgesehen ist.

Hier liegt der Kernpunkt: „On-Zeit“ bezeichnet die Zeit im unmittelbaren Patientenkontakt – Zeit, in der MIT dem Patienten gesprochen wird. „Off-Zeit“ umfasst die Zeit, in der ÜBER Patienten gesprochen wird (Teamsitzungen, Supervisionen), sowie administrative Aufgaben und Fortbildung – allesamt wichtige Tätigkeiten.

Die Herausforderung liegt im Management dieser Zeitverteilung. Die üblichen Erklärungen – Bürokratie, mangelnde Digitalisierung – greifen zu kurz. Natürlich kann durch Entbürokratisierung oder künstliche Intelligenz Zeit freigesetzt werden. Aber was nützt das, wenn diese Zeit wieder mit anderen Aufgaben gefüllt wird? Ohne Orientierungswerte und Monitoring führt zusätzliche Zeit nicht automatisch zu mehr Patientenkontakt.

Appelle an die Politik verhallen oft oder benötigen lange Zeiträume für die Umsetzung. Das Management der vorhandenen Ressourcen hingegen liegt in unserer eigenen Hand und lässt sich rasch umsetzen.

## Bewährte Maßnahmen

Die nachfolgenden Maßnahmen haben sich in unserer Klinik bewährt und die Patientenkontaktzeit deutlich gesteigert. Wir können geeigneten Patienten eine hohe Therapiedichte entsprechend der Kategorie A7 anbieten – bei gleicher Personalausstattung wie vergleichbare Kliniken. Das Ergebnis: Patienten sind besser versorgt und Mitarbeiter haben mehr Zeit für das, wofür sie ausgebildet wurden – die therapeutische Arbeit.

### Transparenz

Systematische Erfassung der erbrachten Einzeltherapien. Nur bei Klarheit über die tatsächliche Leistungserbringung kann auch gesteuert werden.

### Orientierungswerte

Eine On-Zeit von 50–70 % als Zielwert, abhängig von der Fallführerschaft. Dies lässt ausreichend Raum für wichtige Off-Zeit wie Supervision, Intervision und Teamsitzungen. Es geht um eine ausgewogene Balance, nicht um einseitige Maximierung.

### Teilzeitregelungen

Die Teilnahme an Off-Zeit-Terminen muss an die Arbeitszeit angepasst werden. Würden Halbtageskräfte an allen Besprechungen teilnehmen, ginge dies überproportional zulasten der Patientenkontaktzeit.

### Koordinierte Planung

Langfristige Abwesenheitsplanung bei Urlaub und Fortbildung stellt eine kontinuierliche Patientenversorgung sicher.

### Ressourcenschonende Visiten

Fachärztliche Visiten in kleinen Gruppen statt Großgruppenvisiten sind effektiver und ressourcenschonender.

### Aufgabendelegation

Medizinische Fachangestellte unterstützen bei Administration, Blutentnahmen, Therapieplanung und der Durchführung von Therapien wie repetitive transkranielle Magnetstimulation. Diese Entlastung schafft Freiräume für den direkten Patientenkontakt.

### Schutz der Therapiezeit

Ergänzend zu bewährten Maßnahmen erproben wir in einem Pilotprojekt Strategien gegen telefonische Störungen während der Therapie.

## Empfehlungen

### Zwei Handlungsebenen

Die Verbesserung der Versorgung erfordert Maßnahmen auf zwei Ebenen. Erstens können Kliniken durch On-Zeit-Optimierung mit vorhandenen Ressourcen eine erheblich bessere Versorgung erreichen. Zweitens erfordert leitliniengerechte Behandlung eine Erhöhung der Personalvorgaben [7]. Beide Ebenen sollten parallel verfolgt werden.

### Weiterentwicklung der PPP-RL

Trotz formaler Übererfüllung der Mindestpersonalausstattung bleibt die psychotherapeutische Versorgung quantitativ hinter dem Bedarf zurück – ein Hinweis, dass Strukturqualität allein keine Prozessqualität garantiert. Die PPP-RL könnte um Orientierungswerte zur Patientenkontaktzeit ergänzt werden – ein Wert von 50–70 % wäre realistisch. In einem „sprechenden Fach“ geht es nicht nur um Anwesenheit, sondern darum, dass tatsächlich mit Patienten gesprochen wird. Dies wäre ein Schritt von der Struktur- zur Prozessqualität.

### Verantwortung der Klinikleitungen

Die Diskrepanz zwischen objektiven Versorgungsdaten und subjektiver Wahrnehmung auf Leitungsebene zeigt Handlungsbedarf. Die Umsetzung der beschriebenen Maßnahmen hat sich bewährt und ist ohne zusätzliche Ressourcen möglich.

### Patientenwohl als ethischer Standard

Die evidenzbasierte Versorgung schwer kranker Patienten erfordert ausreichend häufige Psychotherapie. Dies ist nicht nur eine Frage der Effizienz, sondern auch eine ethische Verpflichtung. Wenn wir wissen, dass häufigere Einzelgespräche bei schwerer Depression wirksamer sind, sollten wir die organisatorischen Voraussetzungen schaffen, um diese Therapieform bestmöglich anzubieten. So wird der Blick von der Strukturqualität über die Prozessqualität zur Ergebnisqualität gelenkt.

## Fazit für die Praxis

- Stationäre Patienten erhalten nur 25 min/Woche Einzelpsychotherapie.
- Für eine leitliniengerechte Behandlung (100min/Woche bei Depression) wäre eine Personalverdopplung erforderlich.
- Durch On-Zeit-Management ist eine erheblich bessere Versorgung mit vorhandenen Ressourcen möglich.
- Bewährte Maßnahmen: Monitoring, 50–70 % Patientenkontaktzeit als Orientierungswert, angepasste Teilzeitregelungen, koordinierte Planung, ressourcenschonende Visiten.
